# Evaluation of the Serum Zinc Level in Erosive and Non-Erosive Oral Lichen Planus

**Published:** 2014-06

**Authors:** N. Gholizadeh, M. Mehdipour, Sh. Najafi, A. Bahramian, Sh. Garjani, H. Khoeini Poorfar

**Affiliations:** a Dept. of Oral Medicine, School of Dentistry, Tehran University of Medical Sciences, Tehran, Iran.; b Dept. of Oral Medicine, School of Dentistry, Shahid Beheshti University of Medical Sciences, Tehran, Iran.; c Dept. of Oral Medicine, School of Dentistry, Tabriz University of Medical Sciences, Tabriz, Iran.; d Dept. of Oral Radiology, School of Dentistry, Tabriz University of Medical Sciences, Tabriz, Iran.; e Dept. of Pediatric Oncology, School of Medicine, Hamadan University of Medical Sciences, Hamadan, Iran.

**Keywords:** Lichen Planus, T helper, Zinc

## Abstract

**Statement of Problem:** Lichen planus is a chronic inflammatory immunologic-based disease involving skin and mucosa. This disease is generally divided into two categories: erosive and non-erosive. Many etiologic factors are deliberated regarding the disease; however, the disorders of immune system and the role of cytotoxic T-lymphocytes and monocytes are more highlighted. Zinc is an imperative element for the growth of epithelium and its deficiency induces the cytotoxic activity of T-helper2 cells, which seems to be associated with lichen planus.

**Purpose: **This study was aimed to evaluate the levels of serum zinc in erosive and non-erosive oral lichen planus (OLP) and to compare it with the healthy control group to find out any feasible inference.

**Materials and Method:** A total of 22 patients with erosive oral lichen planus, 22 patients with non erosive OLP and 44 healthy individuals as the control group were recruited in this descriptive-comparative study. All the participants were selected from the referees to the department of oral medicine, school of dentistry, Tabriz University of Medical Sciences. Serum zinc level was examined for all the individuals with liquid-stat kit (Beckman Instruments Inc.; Carlsbad, CA). Data were analyzed by adopting the ANOVA and Tukey tests, using SPSS 16 statistical software.

**Results:** The mean age of patients with erosive and non-erosive LP was 41.7 and 41.3 years, respectively. The mean age of the healthy control group was 34.4 years .The mean serum zinc levels in the erosive and non erosive lichen planus groups and control groups were 8.3 (1.15), 11.15 (0.92) and 15.74 (1.75) μg/dl respectively. The difference was statistically significant (*p*< 0.05).

**Conclusion:** The serum zinc levels were decreased in patients with erosive oral lichen planus. This finding may probably indicate the promising role of zinc in development of oral lichen planus.

## Introduction

Lichen planus is a chronic immunologic-based inflammatory disease with mucocutaneous involvement. Oral lichen planus (OLP) is divided into two major groups; erosive and non-erosive. The non-erosive lesion is sub-divided to reticular, papular, plaque like and erythematous [1].

To confirm the clinical diagnosis of OLP, presence of reticular and popular elements is indispensible [1]. In patients with erythematous lesions of gingiva, it might be bewildering to find the white reticular and papular elements and gingival lesions may resemble the pemphigoid lesions. In these cases, incisional biopsy is denoted for definitive diagnosis [1].

Many underlying factors are appealed to be related to or associated with the process of this disease [1-2]. Some of these factors include the disturbance in the body immune system and the role of cytotoxic T-lymphocytes and monocytes, as the main element in the pathogenesis of the disease [1]. Several factors can influence T-lymphocytes such as stress, diabetes, hepatitis C, trauma, drugs and metal sensitivity [2]. Trace elements, such as zinc and copper, are directly involved in metabolic processes that are critical for cell differentiation and replication. Alterations in the level of these elements are considered as a part of defense strategies of organisms which is crucial for stability of cell membrane, apoptosis, host metabolism and enzyme activities [3].

Zinc is one of the most imperative elements in growth and development of epithelium; moreover, it is a requisite element for cellular function and metabolism of carbohydrates, proteins and lipids [3-4].

A slight deficiency of this element leads to decrease in thymulin function and the interleukin2 (IL-2) secretion [4]. Deficiency of zinc leads to imbalance in T- helper lymphocytes (TH), the decrease in the number of TH1 lymphocytes will result in increasing TH2 as the reimbursement process .The increasing in the TH2 function is related to the formation of lichen planus, increasing cytotoxicity and lichen planus pathogenesis [5]. The findings of the Arora et al.’s study indorse the role of zinc deficiency in psoriasis, acne vulgaris and leprosy [6]. Khademi et al. [7] have also substantiated the lower serum zinc level in patients with recurrent aphthous stomatitis.

Regarding to the few experiments performed earlier on other diseases, the current study aimed to assess the level of serum zinc in erosive and non-erosive oral lichen planus to find out any conceivable inference.

## Materials and Method

A total of 44 patients with OLP (22 erosive and 22 non-erosive) and 44 healthy individuals (control group) were recruited in this descriptive-comparative study. 

The study was approved by ethics committee of Tabriz Medical University. All participants were selected from the natives referring to the department of oral medicine, Faculty of Dentistry, Tabriz University of Medical Sciences, Iran.

The participants being 18-60 years old and having oral lichen planus were included in the study. The diagnosis of the non-erosive and the erosive lichen planus was based on clinicopathological features of the disease. To establish a clinical diagnosis of OLP; reticular or popular textures should have been present. Besides, in the histopathologic sections; basal cell degeneration and infiltration of inflammatory cells like T-lymphocytes should have been observed. The exclusion criteria were considered to be:

1. The presence of any factor related to the lichenoid reaction such as amalgam fillings near the lesion and consumption of medications which are associated with lichenoid reaction.
2. The presence of acquired and congenital immunodeficiency disorders like AIDS, chemotherapy, addiction to injectable opioids, hemophilia and blood dialysis. The reason for excluding these patients from our study was the difficulty in their biopsy procedure, control of infection and the possible interaction with clinical findings of lichen planus and their potential doubtful cooperation.
3. Patients whom their histopathologic finding indicated dysplasia.
4. Impassiveness for being involved in the study.
5. Consumption of drugs which influence the serum zinc level, such as zinc acetate, zinc sulfate or other supplements that contain zinc element.
6. Consumption of medication for treatment of lichen planus in the past two months.
7. Patients with malabsorption problems.
8. Presence of factors that alter the zinc absorption like consumption of calcium tablets and iron supplements, pregnancy and lactation phase, as well as the complication that leads to albumin production (high protein diets).
9. Patients in post-surgical acute phase.
10. Alcoholic patients.

The patients’ records were completed and necessary examinations were performed, the biopsy was then taken from all of the patients. The participants were informed about the nature of the survey in details and were asked to read and sign a written informed consent. Subsequently, 3 mL of blood samples were taken from both control and case groups and serum zinc level test was performed by manual method, employing the liquid-stat kit (Beckman Instruments Inc.; Carlsbad, CA). Then statistical data obtained from our research were analyzed by adopting independent sample t-test through SPSS.16 software. The difference of the serum zinc level between the test group and the control group was statistically significant (*p*< 0.05).

## Results

A total of 22(50%) patients with erosive lichen planus and 22 (50%) patients with non-erosive and 44 healthy individuals were studied.

Of the total participants, 53 (60.2%) were female and 35 (39.8 percent) were male. The mean age of patients with erosive LP, non-erosive LP and the healthy control group was 41.7, 41.3 and 34.4 years, respectively.

The statistical two-sample t-test was employed to compare the serum zinc level in patients with erosive and non-erosive lichen planus. The results showed there was a difference in average serum zinc level between two groups (erosive and non-erosive) and the results were statistically significant (*p*< 0.05).

The average serum zinc level in erosive type was lower than the non-erosive type. To compare the serum zinc level of patients with lichen planus and the healthy participant, One -way ANOVA test was performed. The results declared the difference in the average serum zinc level between these three groups (erosive patients, non-erosive patients and healthy individuals) was statistically significant (*p*< 0.05) (Table 1).

**Table 1:** Average and Standard Deviation of measured zinc level in the 3 groups

**Groups**	**(Average ± Standard Deviation)**	**Minimum**	**Maximum**
Erosive	83±(1.15)	6	10.2
Non-Erosive	11.15±(0.92)	9	12.5
Control	15.74±(1.75)	12.4	19
Total	12.75±(3.5)	6	19

## Discussion

The results of statistical analysis revealed a significant difference in average serum zinc level between patients involved with the LP and healthy individuals. The average serum zinc level was decreased in erosive LP compared to the non-erosive LP. 

There are two justifications for the possible effect of serum zinc level on the occurrence of OLP. One of the impacts of zinc level is exhibited in the regeneration of epithelium. Furthermore, zinc enhances the enzyme activity, contributes to protein structure, and regulates gene expression [8].

Zinc is a cofactor for polymerases and proteases involved in many cellular functions such as wound repair and intestinal epithelial cell regeneration [9-10].

Zinc is believed to interact with taurine and vitamin A and has antioxidant effects which may protect macular degeneration caused by oxidative stress [11]. The same effect may be considered in the process of the cellular degeneration caused by LP.

Zinc deficiency is associated with impaired wound healing and the other effect is inhibition and stimulation on lymphocyte reaction.

Thymic epithelial cells secret thymic hormones that have an impact on the maturation of T- lymphocytes.

One of these peptides, thymulin, requires zinc as a cofactor and in an equal molarity ratio for biological activity [10]. The same influence on T-lymphocytes may perhaps be concerned in the process of immunologic-based diseases like LP.

The pathogenesis of LP is not completely recognized. An immunopathological pathogenesis with T-lymphocytes directed against basal keratinocytes or the basal membrane zone is implied for the possible pathogenesis of the LP [11, 12]. The effects of zinc on the immunological responses are promising [13]. The T- lymphocyte signal transduction pathway encompasses several zinc-finger proteins. The immune system is intensely impaired by zinc deficiency and the cell-mediated response by T-lymphocytes is affected mainly [13]. In zinc deficiency conditions, the function, development and the polarization of T-lymphocytes into effectors are disrupted. This process leads to reduction in T-cell numbers, decreased ratio of type 1 /type 2 T-helper cells (with reduced production of T-helper type 1 cytokines like interferon-gamma) and compromised T-cell mediated immune defense [13]. Consequently, disrupted zinc homeostasis increases the risk for infections while zinc supplementation restores normal immune function [12-13]. Adequate zinc level in serum is essential for T-cell division, maturation and differentiation, lymphocyte response to mitogens, programmed cell death of lymphoid and myeloid origins, gene transcription, and biomembrane function [12-13].

There is a limited study available reporting the zinc level in OLP, therefore, the findings of surveys performed on the lesions similar to OLP is compared with the results of this study.

The results of this study are consistent with the findings of Arora et al.^’^s study that confirmed the role of zinc deficiency   in certain diseases such as psoriasis, acne, vulgaris and leprosy [6]. Since lichen planus is a mucocutaneous disease and zinc has a substantial role in development of epithelium, ulceration in dermatologic diseases and simultaneous zinc deficiency in these diseases may endorse the role of this element on pathogenesis of lichen planus disease.

No significant alterations in the serum zinc levels were detected in cases of vitiligo and aphthous ulcers, compared to healthy individuals in the study of Arora et al. [6]. It seems that the reason for this difference is the dissimilarity in type of involved autoimmune disease and the type of aphtus lesions (minor type) in their study which apparently could not influence the serum zinc level. The exact time of the disease process in which the zinc level is evaluated is not clear in the Arora et al. research. Since aphtus ulceration (like lichen planus) is a disease with immunological contributions, the role of zinc in its pathogenesis is under debate. A study on zinc measurement was carried out by Shameer et al. [14]. They stated that serum zinc level in the patient with vitiligo was less than healthy individuals in the control group [14]. The etiology of vitiligo is unknown, but studies suggest it may arise from autoimmune, genetic and oxidative causes. Interleukin-1β is expressed at high levels in patients with vitiligo that may substantiate the proposed etiologic factors [15].

In a study enrolled by Agren et al., topical zinc was used in wound treatment and they concluded that post-operative serum-zinc levels increased (*p*< 0.001) in both experimental and placebo groups but did show any significant difference between the two groups on day seven [16].

Mehdipour et al. surveyed the benefits of topical application of zinc [17]. They concluded that zinc sulfate mouthwash (0.2%) containing fluocinolone and mouth washes (having fluocinolone alone) were both effective in decreasing the associated pain and irritation and also the surface area of OLP. They confirmed that decrease in surface area with zinc-plus-fluocinolone mouthwashes were more than that observed in cases treated by mouthwashes containing fluocinolone alone [17]. The relative association of the zinc with the nature of the OLP may be assumed from the findings of their study which is almost in line with the findings of the current study including a link between serum zinc level and OLP.

Zinc deficiency is also claimed to be in association with impaired wound healing and it may increase the re-epithelialization [18]. These findings may also weigh up the possible relationship between the serum zinc level and the character of the OLP.

## Conclusion

Within the limitations of this study, Serum zinc levels were found to be significantly reduced in patients with erosive OLP compared to non- erosive OLP. Further studies with large number of samples are entailed to confirm this finding.
